# Digital Footprints in the Video Stream: Survey study of reflections on digital traces of media consumption and potential to use this for insights into well-being

**DOI:** 10.23889/ijpds.v8i3.2281

**Published:** 2023-09-11

**Authors:** Joanne Parkes, Giovani Schiazza, Sarah Martindale, Richard Ramchurn, Andrew Smith, Steve Benford

**Affiliations:** 1 N/LAB, NUBS, University of Nottingham; 2 School of Computer Science, University of Nottingham; 3 School of Computer Science, University of Nottingham; 4 Department of Cultural, Media and Visual Studies, University of Nottingham

## Introduction & Background

Netflix now has a consumer base of over 230 million worldwide. During the pandemic, its customers watched 203.8 million hours of content daily, with their activity, content choices and preferences being continually logged. The digital footprint data amassed in this process underpins a symbiotic relationship between supplier and consumer. Black-box algorithms convert these logs into personalised functionality and recommendations, producing improved customer experiences while generating revenue for the business. Whether the consumer willingly accepts this trade-off or not, it’s now almost impossible to use online services without leaving digital traces. But how representative of an individual’s actual preferences and behaviours are these? What biases exist in such datasets? And to what degree are consumers cognisant of how these datasets are being used?

## Objectives & Approach

This study surveyed participants to interrogate their understanding of the data Netflix makes available to its subscribers. The objectives were to explore their perceptions relating to the data collected about them and encourage them to think critically about their digital footprint. It was also the intention of the research group that participants feel a sense of empowerment / control over the data made available to them.

UK-based participants were provided with instructions on how to access their viewing history (programme titles, dates of access) and invited to inspect it. 61 participants opted to donate their data to the study, along with responses to a survey reflecting their understanding of what they had retrieved.

## Relevance to Digital Footprints

While it may have been possible to work with Netflix to retrieve viewer data, by accessing via the participants instead, the researchers were enabling them to review and make informed choices about what they shared. One of the potential issues with this approach is that it provides an opportunity for participants to curate their data, should there be content that they would be uncomfortable sharing. Alternately, they may choose to withdraw from the study altogether based on what they see. While this has its drawbacks in terms of data inaccuracies and self-selection effect, it was felt important to the research team to prioritise the participants autonomy, encouraging them to be candid and share. If nothing else, it is hoped that by taking part in the study, there is the potential for participants to be inspired to think about the footprints they leave every time they go online so that they might be more mindful of them in future.

## Results

In terms of bias, using only the Netflix data meant that the researchers were only accessing participants who pay for that service. Further, the researchers would only be accessing what would be a proportion of the participants’ viewing. If only using one service however, Netflix is arguably the service to use as according to statistica_©_, In 2021 it was the most subscribed (paid) supplier in the UK.

76% of respondents view more streamed content than terrestrial broadcast content and utilise an average of 3.5 streaming services. 36% of respondents also stated that they share their Netflix user profiles with at least one other person. Despite these limitations, 84% of respondents nonetheless considered that the captured content was representative of their ‘personal tastes and viewing habits.’

76% were not aware until participating in the study that it was possible to extract their viewing data from Netflix, and 34% said they’d likely review it again. 33% indicated surprise as to the extent of information captured about them; but 91% believed that the streaming platform collected more information than was made available.

## Conclusions & Implications

This study shows the potential of data donation to understand viewing habits, binge watching and related well-being indicators, with 43% of surveyed individuals offering their data for research.

What has not been established in this study is why 57% of the group declined to share their data. It can be speculated that it may have been a reluctance to share once the data was inspected or that the process to access and then upload it may have been too much of a hurdle. An implication for this type of study may include a requirement to over-recruit in anticipation of a high drop-out rate or that data extraction and sharing needs to be made as simple and convenient as practicable for the participant.

Given that one of the objectives of the research was to encourage participants to have more curiosity in and awareness / control of their digital footprints, consideration should be given to seeing if participant interest in further exploration of their data could be increased from the 24% seen here. This might be driven by the data type, any perceived utility it might have for the participant or any perceived ways in which it might be used to impact / influence them in some way by a 3rd party.

